# Seal and Sea lion Whiskers Detect Slips of Vortices Similar as Rats Sense Textures

**DOI:** 10.1038/s41598-019-49243-5

**Published:** 2019-09-05

**Authors:** Muthukumar Muthuramalingam, Christoph Bruecker

**Affiliations:** School of Mathematics, Computer Science and Engineering, City, University of London, London, UK

**Keywords:** Sensory processing, Fluid dynamics

## Abstract

Pinnipeds like seals and sea lions use their whiskers to hunt their prey in dark and turbid situations. There is currently no theoretical model or hypothesis to explain the interaction between whiskers and hydrodynamic fish trails. The current study, however, provides a theoretical and experimental insight into the mechanism behind the detection of the Strouhal frequency from a Von-Karman vortex street, similar to that of the inverted hydrodynamic fish trail. Herein the flow around a 3D printed sea lion head, with integrated whiskers of comparable geometry and material properties to a real seal lion, is investigated when exposed to vortex streets generated by cylindrical bluff bodies. The whiskers respond to the vortices with a jerky motion, analogous to the stick-slip response of rat whiskers; this motion is found to be the time derivative of the Gaussian function. Compared to the displacement response, the time-derivative of the whisker response decodes the Strouhal frequency of the Von-Karman wake, which improves the sensing efficiency in noisy environments. The study hypothesizes that the time derivative of the whisker bending moment is the best physical variable that can be used as the input to the pinnipeds neural system.

## Introduction

Nature has provided sensing capabilities for different living organisms depending on their living environment and the struggles they encounter in their habitats. For instance, fish use their lateral line system to detect even the slightest movement in the water^1^; crocodiles use miniature sets of dome-shaped pressure sensors to perceive the movement of their prey^2^. Bats, whales, and dolphins use the bio-sonar system to locate the objects and recognize them^3,4^. Similarly, land mammals such as rats and squirrels vibrate their whiskers, commonly known as ‘whisking’ to obtain data about their surroundings^5^. Pinnipeds like Phocidae and Otariidae also have highly sensitive whiskers that function as hydrodynamic receptors, which can detect even minute velocity changes in the water generated by fish.

Dehnhardt *et al*. demonstrated that Harbor seals respond to dipole stimuli ranging from 10–100 Hz with a minimum perceivable water velocity of 0.25 mm/sec at 50 Hz^6^. Later, Dehnhardt found that Harbor seals could track hydrodynamic trails generated by a model submarine even after a period of 25 sec, which corresponds to a chase length of 40 m^7^. However, Gläser *et al*. performed the same experiment as Dehnhardt with a California sea lion rather than the Harbor seal. They found that the California sea lion can also follow the wake trail, but it could only track the model submarine if the delay time was 7 sec or less^8^. In dipole experiments, California sea lions are even more sensitive than Harbor seals. They have already responded to sinusoidal water motions with a velocity amplitude of only 0.35 mm/sec in the frequency range 20 to 30 Hz^9^. The differences in tracking ability between these two species are ascribed to the whisker’s topological variation. Harbor seal whiskers have the form of tapered beams with an undulating elliptic cross-section. This undulating geometry was seen to reduce self-excited vibrations or ‘Vortex Induced Vibrations’ (VIV)^10^. The VIV phenomenon is induced by alternating shedding of vortices from either side of the whisker cross-section, resulting in a strong periodic force variation in cross-flow direction (see Appendix-1 for comprehensive description). On the other hand, the California sea lion whisker has an elliptical cross-section that tapers towards the tip and has a constant perpendicular orientation over the length^11^. This geometry was observed to not perform well in reducing VIV. Miersch *et al*. compared the hydrodynamics over the isolated whiskers of the two species in a rotating flow tank and quantified that Harbor seal whisker’s signal to noise ratio (SNR) is ten times greater than that of the California sea lion whisker. This large difference was attributed to the reduced accuracy in wake tracking ability of the species^12^ as observed previously by Glaser *et al*. They added that the sea lion whisker’s self-excited oscillations, linked to VIV, could serve as a carrier signal that can be used to determine the self-swimming velocity. Beem and Triantafyllou studied the flow over model whiskers manufactured of plastic using stereolithography. They described the whisker motion as ‘slaloming’ with the vortices when exposed to vortex streets generated from an upstream cylinder. Compared to the clean flow conditions, the model whisker’s vibration amplitude was very large in the upstream cylinder wake (Referred to Wake-Induced Vibrations (WIV) to differentiate from the self-induced vibrations (VIV)) (Appendix-1). This larger amplitude of the whisker vibration is considered to be the reason for the improved sensitivity of Harbor seals^13^. Schulte-Pelkum has demonstrated that harbor seals can effectively follow the biogenic trails of the same species. Seal pups could use this tracking ability to follow lactating mother seals during feeding time^14^. In this study, the trail-following seal trajectory was aligned with the pilot seal path for most trials. In some of the trials, however, the follower crossed the path in a zig-zag way to redefine its course. Additionally, it was inferred that the trail-following seal was able to resolve the wake’s inner flow structure while tracking.

Wieskotten *et al*. found that the Harbor seal could indicate and follow the direction of a wake trail generated by moving an artificial flexible fin up to a delay of 35 sec^15^. As such, it was suggested that the seal could have used the information in the high-velocity region between the two counter-rotating vortices and the vortex street structure as a hydrodynamic cue to track the direction. In a successive experiment, Wieskotten *et al*. found that the Harbor seal could discriminate the size and shape of the objects by tracking their corresponding wakes^16^. They also concluded, however, that the ability to discriminate in size and shape is based on more than one hydrodynamic parameter. Krüger *et al*. argued that harbor seals use vortex rings as the primary source of directional discrimination^17^. By simulating a range of test cases with single vortex rings, it was concluded that the minimum perceivable angle between the anteroposterior axis and the vortex ring path was less than 5.7°, however, the minimum value was not found due to technical limitations. Niesterok *et al*. experimented with intermittent jets from an underwater surface to imitate artificial flatfish breathing currents on sea lions and found that the seals could detect velocities in the range of 2–2.5 cm/sec^18^. This value is significantly higher than the value (0.25 mm/sec) obtained from dipole studies^6^. Due to the movement of the animal, the self-induced velocity variations could be the cause behind this reduced sensitivity. Murphy *et al*. tagged an accelerometer on a supraorbital whisker of a true Harbor seal and observed that if exposed to an upstream hydrodynamic disturbance, the seal whiskers vibrate over a broad range of frequencies^19^.

Sensors based on mammalian whiskers were developed and tested successfully in biomimetic applications. Subramaniam *et al*. tested a 3D printed individual Harbor seal whisker in a towing tank and reported that Harbor seal whisker’s VIV is lower in magnitude compared to cylindrical whisker^20^. Solomon and Hartmann used multiple tandem whiskers to trace and reconstruct complex surfaces using Euler-Bernoulli beam theory as well as measuring the velocity profile in a free jet flow^21^. Inspired by the seal’s whisker array, Eberhardt *et al*. placed eight cylindrical whiskers on a torpedo-shaped body to sense the wake interference from an upstream submarine model^22^. They concluded that the use of multiple whiskers will assist in accurate object tracking and can be used in surveillance.

Despite all the earlier studies still some fundamental questions remain unanswered in the study of pinnipeds and their hydrodynamic trail tracking abilities. To list a few, what impact does the animal’s head have in changing the signal strength, do they make use of multiple whiskers and how the arrangement of multiple whiskers interacts with oncoming alternating vortices from wakes generated by fish. Fish swim at cruising conditions within a very narrow Strouhal number band, St $\simeq$ 0.2 for a broad range of Reynolds number^23^. The Strouhal number and the Reynolds number are defined by equations 1 and 2, where ‘f’ is the tail-beating frequency or the Strouhal frequency, ‘D’ is the length scale of the fish, ‘U_∞_’ is the swimming speed and ‘$\rho$’ and ‘*μ*’ are the density and viscosity of the fluid (water).

1 $$St=fD/{U}_{\infty }$$

2 $${Re}=\rho {U}_{\infty }D/\mu $$

At cruising, as the Strouhal number is a constant, swimming speed is proportional to the tail-beating frequency^24^. The frequency is, therefore, the single most significant characteristic to identify the fish’s swimming speed. The wake behind a 10 cm long swimming goldfish generated a wake path of measurable velocities for at least 30 sec^25^ and this time-lapse should theoretically scale with the fish’s length. Since Harbor seals feed on fish with an average length of 30 cm^26^, the information left behind the fish wake is certainly a strong hydrodynamic cue for predators. The crucial question here is, how the neural system of the seal decodes this information in an otherwise noisy environment. From a fluid dynamics perspective, when the seal swims with its whiskers protracted forward, the fluid-induced drag forces induce the whiskers to bend in the mean flow direction and hence the swimming speed is directly proportional to the whisker’s deflection (Appendix-2). Since whiskers have no nerves^27^, therefore, the nerve endings in the whisker pads read the information about the bending moment at the root of the whiskers. These nerves connects the somatosensory system in the brain of sea lions^28^. Consequently, a wake trail of a fish swimming upstream should alter the whisker’s bending moment based on the flow-induced force distribution along the whisker and this could be encoded with the neural system. A satisfactory answer to this question is not there in literature for the following reasons: First, it is very difficult to experiment with live seals or sea lions in a regulated environment such as steady swimming speeds without any lateral head motions. Second, it is very challenging to monitor the motion of the whiskers and the vortex convection concurrently in the same experiment when the wake generator is moved upstream. This is the primary objective of this research and it is the first type of experiment on pinniped whisker-like models, which is almost comparable to the realistic scenario.

**Figure 1:**
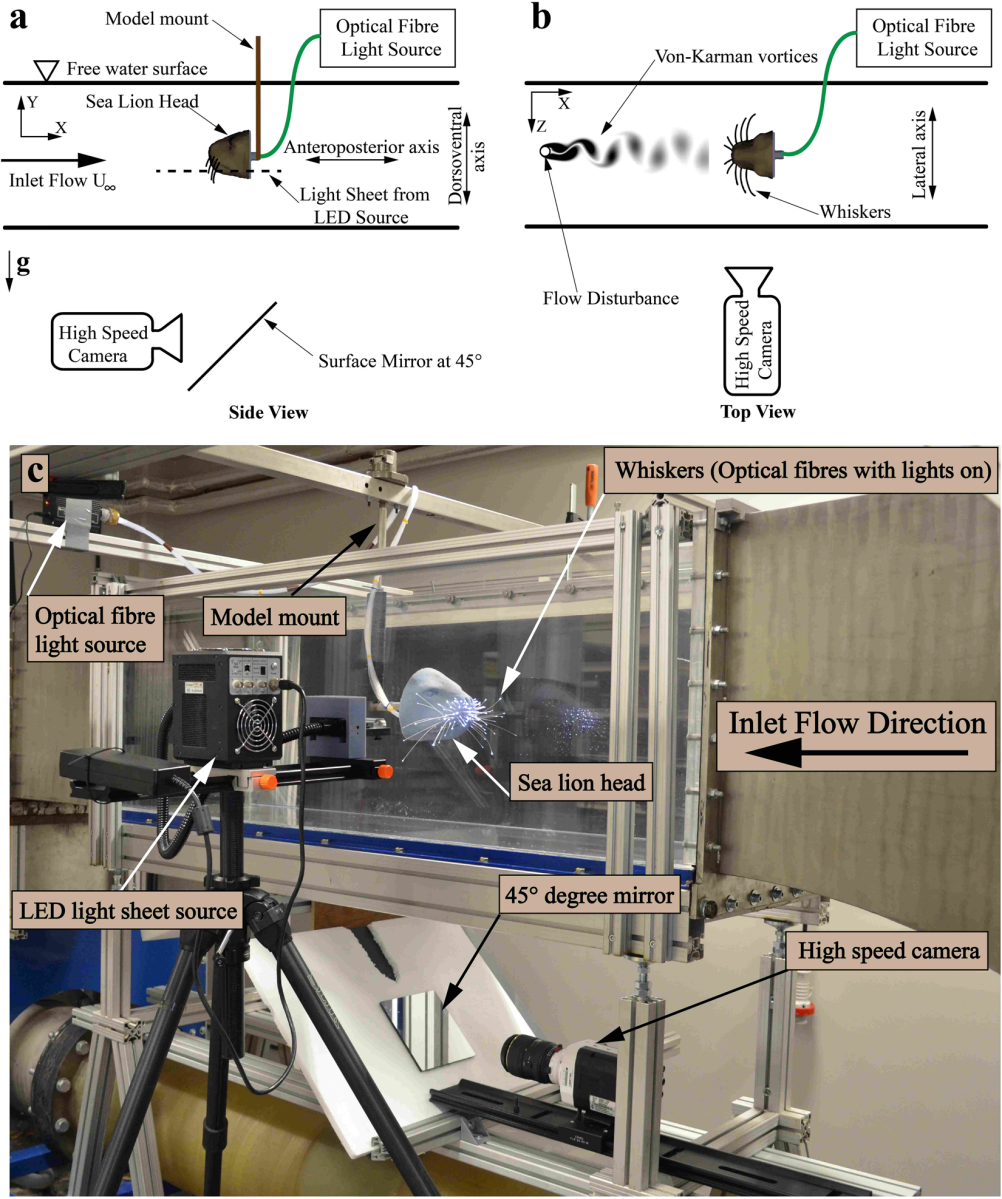
(**a**) Schematic diagram of the experimental set-up (side view). (**b**) Schematic diagram of the experimental setup (top view). Von-Karman vortex street is shown for representation. (**c**) Actual experimental set-up (Note that the tunnel is not filled with water to get a clear view of the model).

The experiments were conducted in a return type open surface water tunnel with a velocity range of 10 to 200 cm/sec. Flow straighteners were installed before the convergent section in the settling chamber to maintain uniform flow in the test section. The test section is 40 cm wide × 50 cm depth × 120 cm in length and transparent in all the sides to provide optical access for flow studies. The sea lion model was placed at 70 cm downstream of the inlet and at the centre of the test section as shown in Fig. 1. Different wake-generating cylinders (modelling the fish wake) were indiviually placed at the inlet of the test section. The experiments were conducted with cylinders of diameter (D) = 25, 28, 30 and 35 mm at three flow velocities (U_∞_) of 20, 25 and 30 cm/sec, where these parameters were seleted based on previous findings^12^. The experiments were divided into two phases. The first phase was to monitor the whiskers’ movement, where, as shown in Fig. 1b, the ‘Eye Motion’ camera focused on just one side of the sea lion model. A subsequent motion tracking experiment was carried out with the ‘Eye Motion’ camera underneath the tunnel (Fig. 1a) in order to observe all of the whiskers simultaneously. The second phase was a study with particle image velocimetry (PIV) to investigate how the shed vortices from the upstream cylinder (see Fig. 1b) interact with the whiskers. It should be noted here that the LED light sheet source used for the PIV study is removed and for motion-tracking measurements, the ‘Eye Motion’ camera was installed at the same location.

## Results

### Wake induced vibrations and vortex induced vibrations

**Figure 2:**
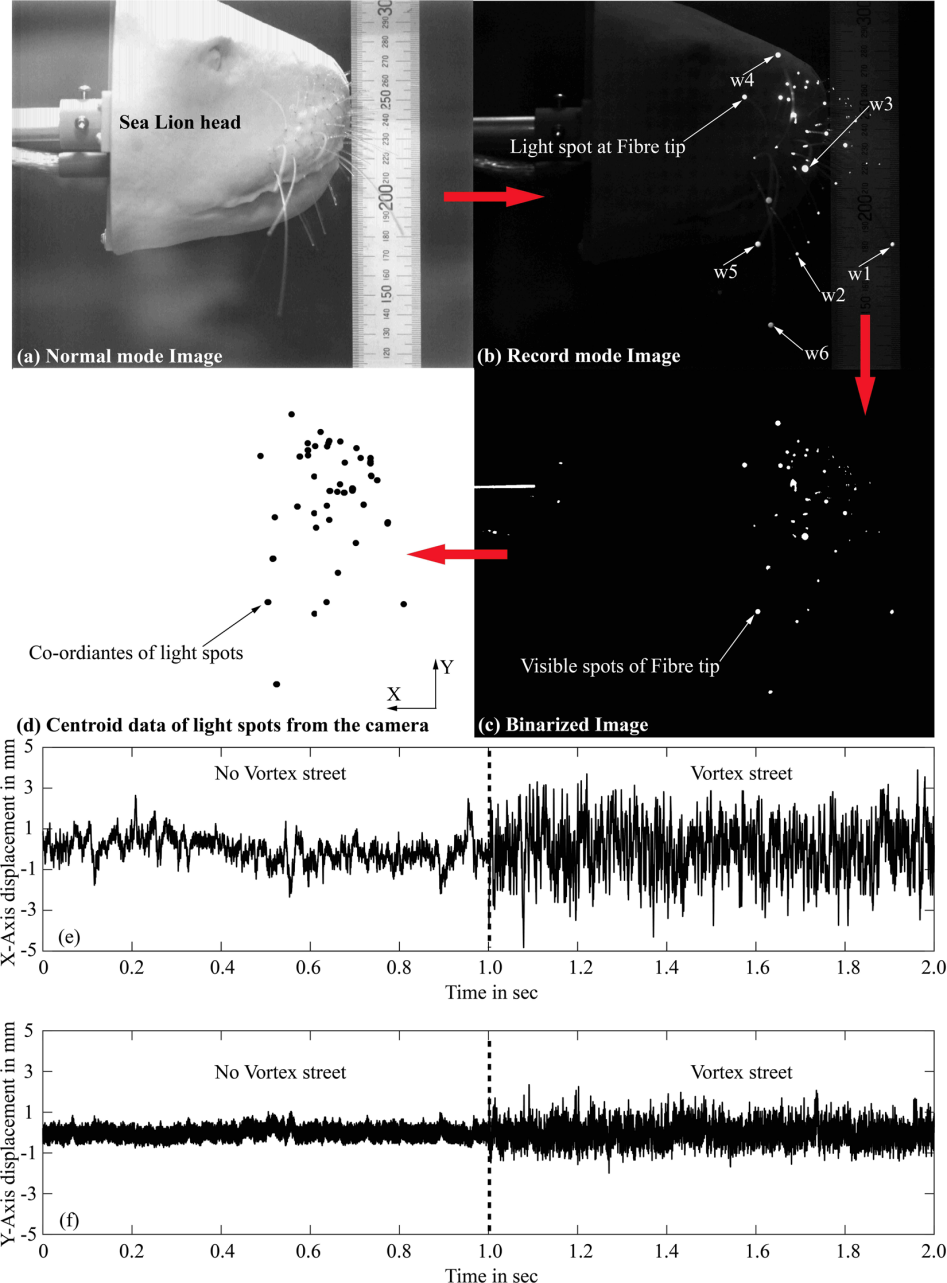
(**a**) Normal mode photograph of the model from the camera (Scale in mm). (**b**) Image from the camera with reduced light and fibre optic light switched ON (the tips of fibres are visible as light spot) Note that the whisker numbering is used in the discussion of results. (**c**) Binarized image in which the fibre tip light spot is converted into white spots greater than certain threshold. (**d**) Coordinates from the light spots in x-y plane. (**e**,**f**) Displacement of whisker tip in streamwise (anteroposterior) and crosswise (dorsoventral) direction with and without vortex street.

The results of the six indicated whiskers in Fig. 2b, are the only ones which will be presented within this study. The whiskers are identified as w1, w2, w3, w4, w5 and w6 in which the whisker w2 has a length of *L*_2_ = 50 mm and all other whiskers were approximately *L*_1,3,4,5,6_ = 100 mm in length. The whisker’s tip motion were tracked in a plane parallel to the X-Y plane as shown in Fig. 1a. The time history of each whisker can be obtained as a coordinate file from the ‘Eye motion’ camera and the data for whisker w3 at 30 cm/sec is shown in Fig. 2e,f. The whisker bending in x-direction is along the anteroposterior body axis i.e. aligned with the mean flow direction. The displacement reported in the results are the instantaneous deflections that are subtracted from the mean deflection. The sign convention is such that a positive displacement is when the whisker moves in the direction of swimming (opposite to the direction of incoming fluid flow in the water tunnel). The displacement in the y-direction is along the dorsoventral axis. When a whisker experiences an upstream wake, the displacement in the streamwise direction (anteroposterior direction) is considerably altered due to WIV rather than the crosswise displacement (dorsoventral direction) that arises due to VIV. As explained in Appendix-2, the tip deflection is a function of the bending moment induced by the corresponding fluid forces in that direction. Therefore, the spectral results along the x-axis correspond to the bending moment spectra and the movements along the y-axis correspond to the VIV spectra.

**Figure 3:**
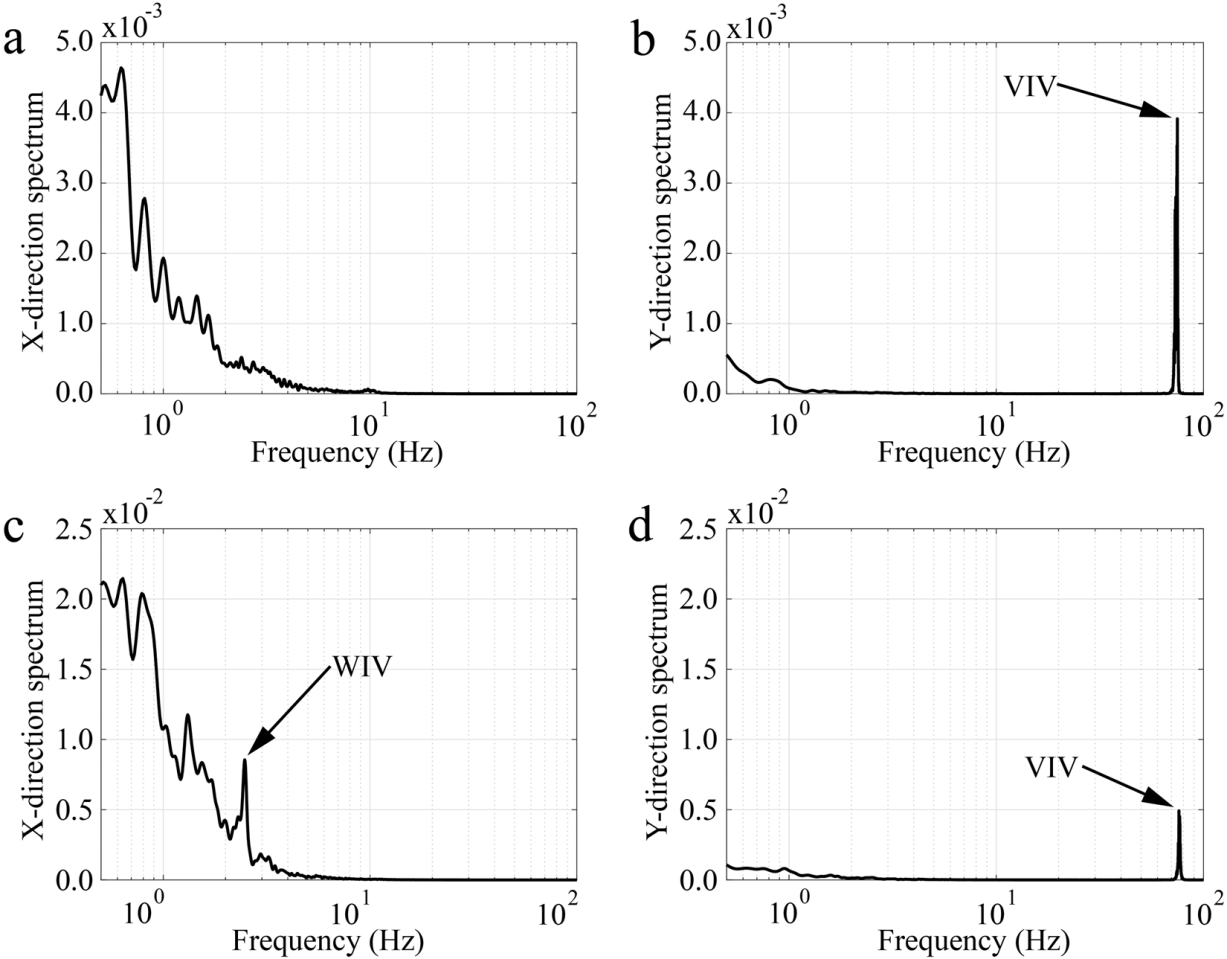
Spectrum of whisker tip fluctuations (whisker3 w3) in undisturbed water flow (**a**,**b**) and when exposed to a Von-Karman vortex street (**c**,**d**). The VIV spectrum is highlighted in the motion along the dorsoventral axis (distinct peak at 75 Hz in (**b**,**d**)) while the WIV (Streamwise bending spectrum) show up in those along the anteroposterior axis corresponding to the swimming direction (distinct peak at 2.4 Hz in (**a**,**c**)).

Figure 3 shows different spectrum of the motion of whisker w3 with and without exposure to a Von-Karman vortex street, in both horizontal and vertical directions at 30 cm/s. When looking at the spectra in the mean flow direction (vibrations along the anteroposterior axis), Fig. 3a, multiple frequencies below 2 Hz can be seen. Conversly, when looing in the vertical direction (vibrations along the dorsoventral axis), Fig. 3b, a strong peak at 75 Hz can be observed. This peak at 75 Hz represents the VIV induced by the alternating shedding of shear layers from the whisker^29^. When calculating the Strouhal number for this based on the diameter of the whisker (d = 0.75 mm) a value of St_*d*_ = 0.19 is obtained and the corresponding Reynolds number based on the diameter of the whisker is Re_*d*_ = 225. At the specified Reynolds number, the reported Strouhal frequency is in good agreement with earlier literature for flow around circular cylinders^30^. It is important to note that the 75 Hz component of the spectrum is the dominant mode of whisker vibration in undisturbed flow (in the absence of any fish wake vortices). When the same whisker experiences a Von-Karman vortex street, generated by placing a 25 mm diameter cylinder upstream of the model, significant changes can be observed in its response. Figure 3c shows the WIV response in the flow direction with a distinct local peak at 2.4 Hz. The Strouhal number based on the 2.4 Hz spectral peak and the upstream cylinder diameter (D = 25 mm) is St_*D*_ = 0.2. This corresponds exactly to the value cited in the literature for a cylinder at a Reynolds number of Re_*D*_ = 7500^31^. An increase in energy at lower frequencies <2 Hz can also be observed, with the appearance of the WIV’s first subharmonic at 1.2 Hz. In addition, noticeable peaks can be observed below 1 Hz, which is believed to result from a low-frequency sloshing motion in the open-surface water tunnel. When looking at the vertical motion (Fig. 3d), the whisker’s VIV frequency is still present in as a distinct peak at 75 Hz. However, the magnitude of this peak is now at least half of the WIV frequency peak in Fig. 3c. This is because the dominant cause for the forces acting on the whisker originates from the upstream wake and its fluctuating fluid motions (WIV dominates VIV). It should be emphasized here that sea lion whiskers with smooth elliptical cross-sections reported a reduction in VIV magnitudes compared to our artificial whiskers with a cylindrical section. The results presented, with cylindrical fibres, indicate that the WIV response is still of higher significance than the VIV response even when such simplified whiskers encounter a wake flow. Therefore, in the natural scenario, the WIV response is even more important for non-cylindrical whiskers.

### Frequency response for 25 mm cylinder at *U*_∞_ = 30 cm/sec

**Figure 4:**
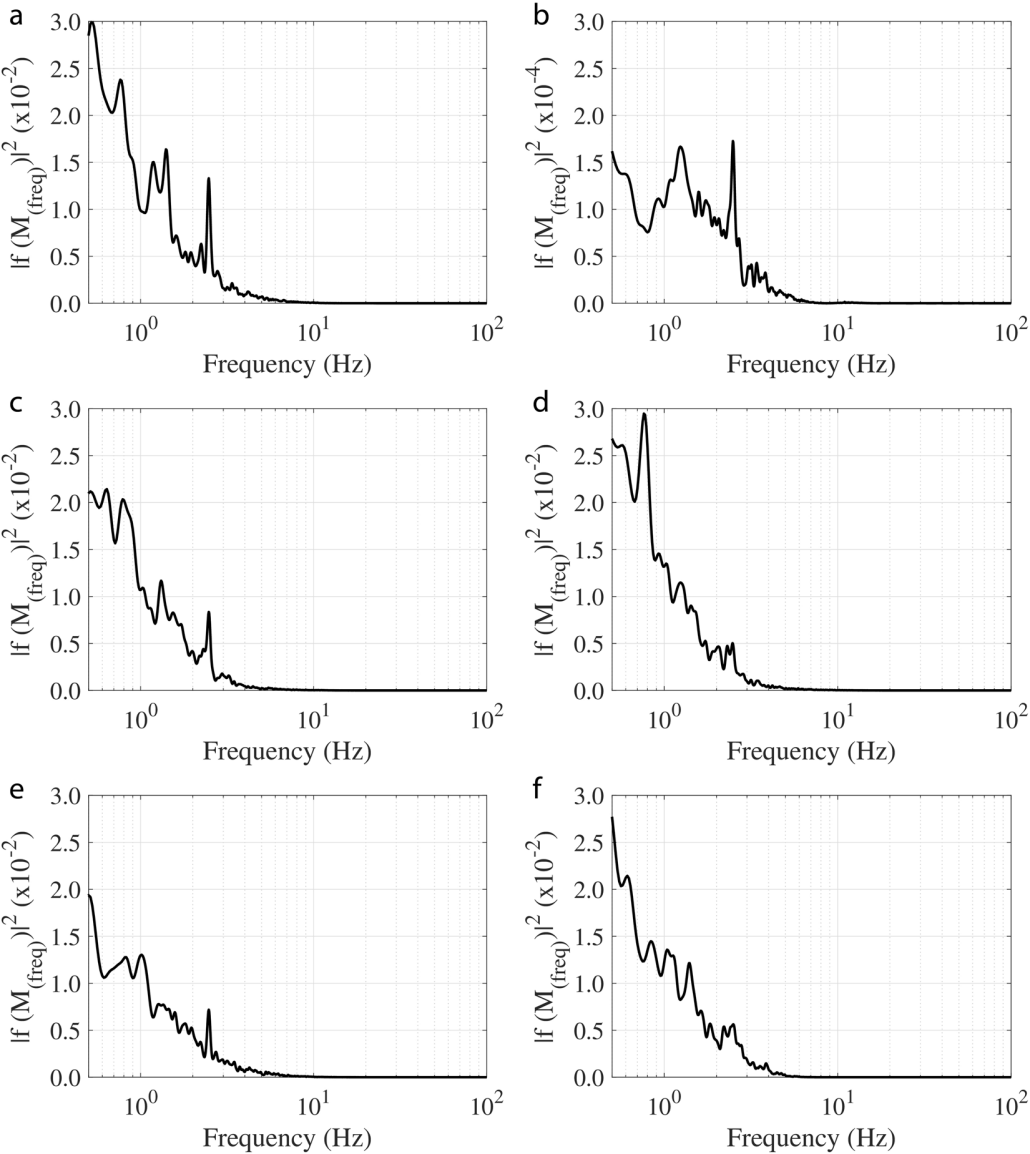
Spectrum of tip fluctuations of different whiskers along the anteroposterior axis when exposed to a Von-Karman vortex street behind a 25 mm cylinder in front ((**a**) w1, (**b**) w2, (**c**) w3, (**d**) w4, (**e**) w5, (**f**) w6). All whiskers are of length L100 mm, except whisker w2 is only of medium length with *L*_2_ ~ 54 mm, therefore note the different axis scale in plot (**b**).

**Figure 5:**
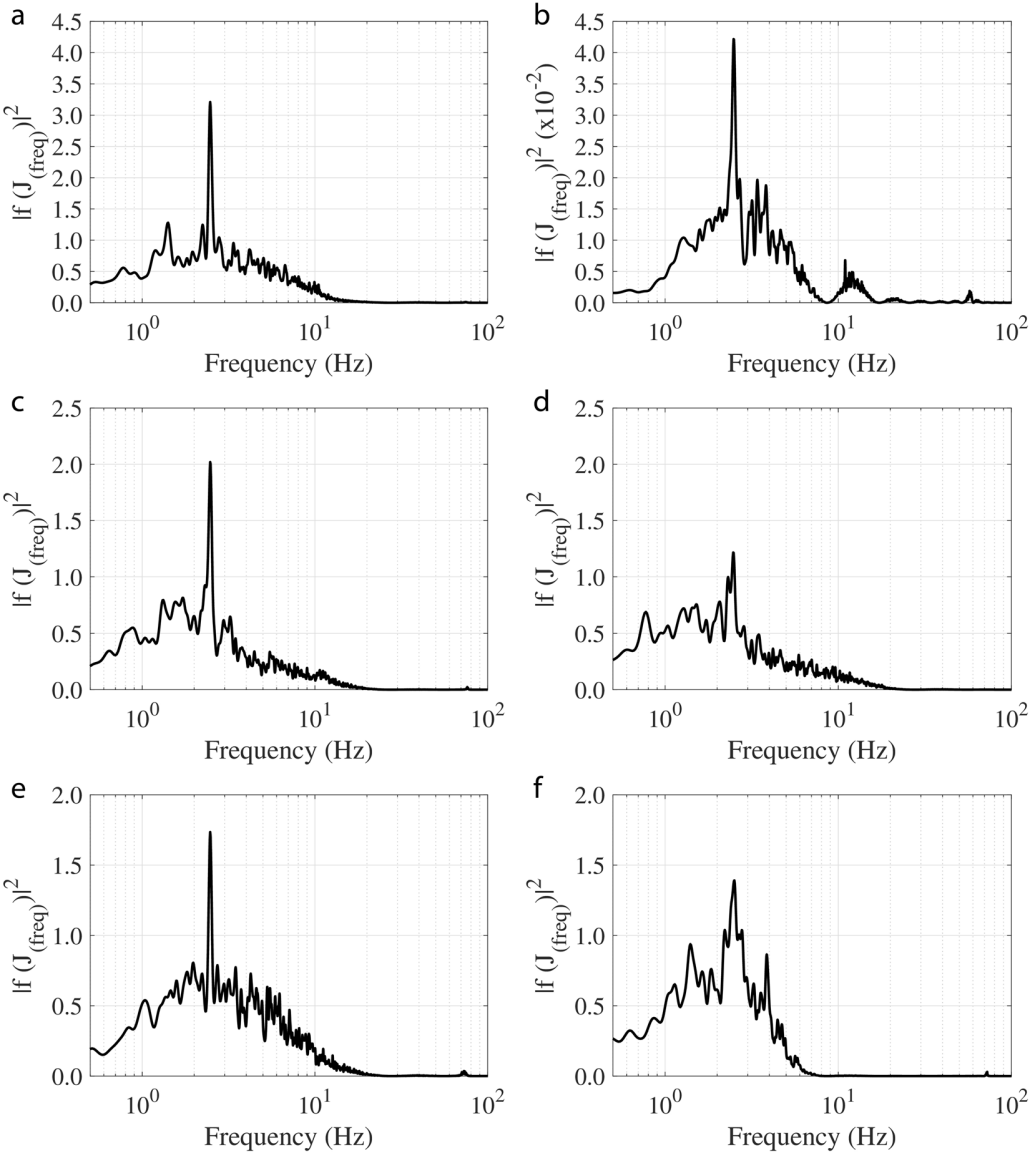
Jerk spectrum of different whiskers along the anteroposterior axis when exposed to a Von-Karman vortex street of a 25 mm cylinder ((**a**) w1, (**b**) w2, (**c**) w3, (**d**) w4, (**e**) w5, (**f**) w6). Note the peak at 2.4 Hz in all six whiskers corresponding to WIV.

The WIV frequency response, calculated from the whisker’s tip displacement in the wake of a 25 mm cylinder with an inlet flow velocity *U*_∞_ = 30 cm/s, is shown in Fig. 4. WIV response relies mainly on the whisker’s orientation, length and position on the head. Figure 4a,c,e indicate a distinct 2.4 Hz WIV peak as previously described. However, compared to the frequency spectrum <1 Hz, this peak is not always dominant. In addition, there is no indication of a strong peak at all in Fig. 4d,f (whisker w4 and w6). Finally, the medium-length whisker (w2 in Fig. 4b) only exhibits this peak, when magnifying the represented axis in Fig. 4b, by about a factor of 100 against the other plots. This highlights that it may not be adequate to differentiate between noise and vortex streets by using solely the bending moment (proportional to the tip displacement, see Appendix-2) as the signal for further neural processing. It could result in an unsuccessful attempt to track the vortex source (prey fish). It should be noted here that the signal strength of the Strouhal frequency could be improved by comparing the left and right whisker pair^32^. This result is comparable to the adaptive filter which is used in the lateral line sensors found in fish to cancel out their self-induced noise^33^. For all six of the tracked whiskers, the spectrum calculated from the time-derivative of the tip-displacement signal is displayed in Fig. 5. A dominant peak at 2.4 Hz is noticeable for all whiskers, which corresponds to the Strouhal frequency of the Von-Karman vortex street generated from the upstream cylinder. The signal to noise ratio (SNR) calculated in the range of 1–5 Hz using the bending moment spectrum is 1.4 (from Appendix-2, the tip deflection is directly proportional to the bending moment) and is 3.9 when using the jerk spectrum. This demonstrates that SNR increases by a factor of approximately 3 by using the jerk spectrum.

### Best frequency analysis for different flow speeds and cylinder diameters

**Figure 6:**
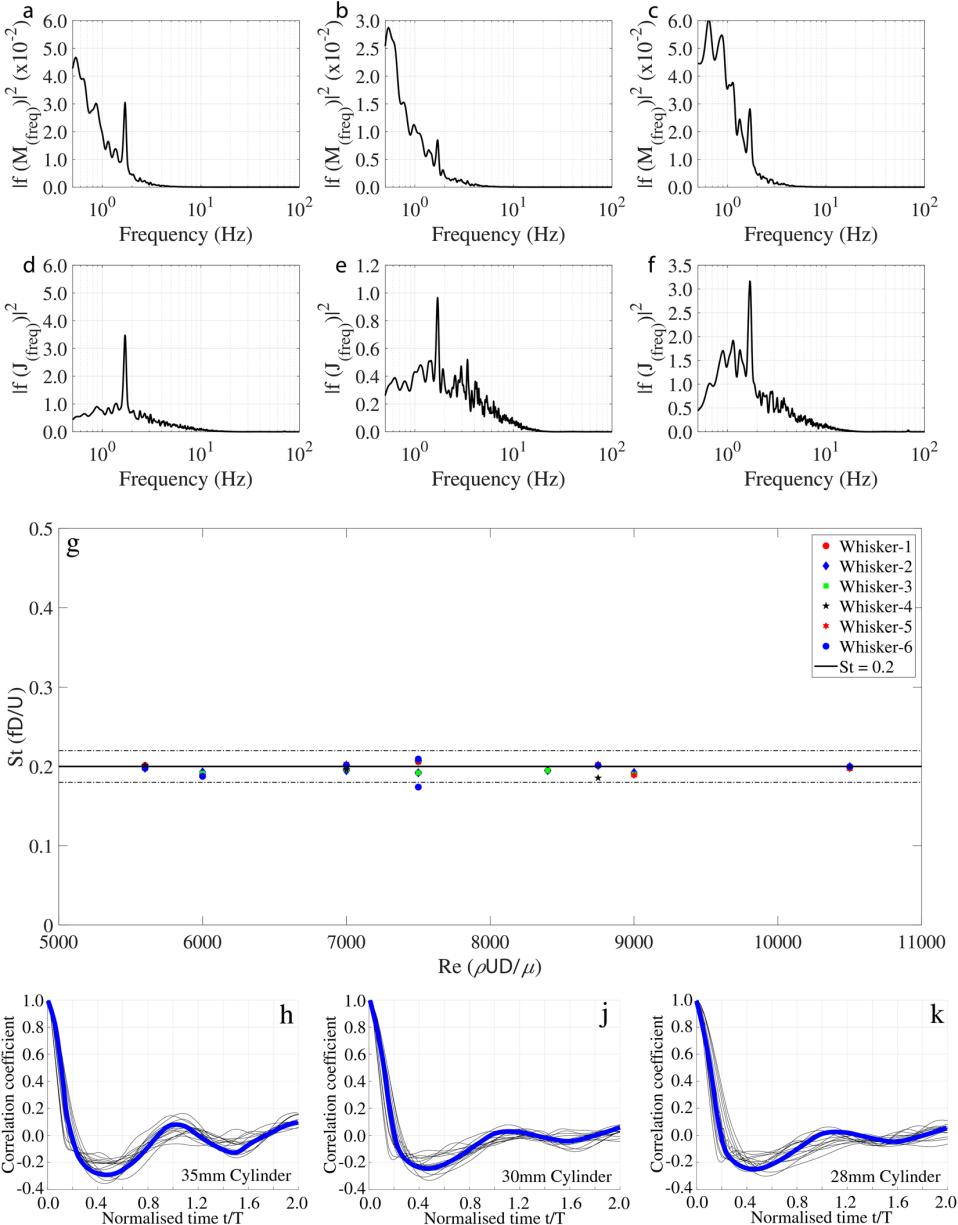
Spectrum of tip fluctuations of different whiskers along the anteroposterior axis for a 35 mm cylinder in front ((**a**) w1, (**b**) w4, (**c**) w5). Jerk spectrum for the same case ((**d**) w1, (**e**) w4, (**f**) w5) (note the peak at 1.7 Hz). (**g**) Reynolds number vs predicted Strouhal number from jerk spectrum for various test cases (**h**,**j**,**k**) Auto-correlation data of jerk signal for cylinders D = 35,30 and 28 mm at all flow speeds.

For the case with a larger cylinder (D = 35 mm) at a flow speed of *U*_∞_ = 30 cm/sec, the frequency response of whiskers w1, w4 and w5 are shown in Fig. 6a–c. Again, at the anticipated WIV frequency (1.71 Hz), the bending spectrum does not indicate a significant peak. Only small peaks appear at 1.71 Hz, corresponding to the Strouhal frequency, and the subharmonic peaks appear at 0.86 Hz. However, the peaks at 1.71 Hz are distinct when the frequency response is plotted with the corresponding ‘jerk’ response, for the same whiskers (see Fig. 6d–f). Thus, it is clear that the best frequency identification method for all whiskers is by using the using the jerk signal, as this clearly reveals the Strouhal frequency generated by the wake of the upstream cylinder. The dominant peak from each of the jerk spectra are then used to calculate Strouhal number (eqn. 1) for all cases considered in this study (D = 25, 28, 30 and 35 mm at flow velocities *U*_∞_ = 20, 25 and 30 cm/sec). The results are shown in Fig. 6g and can be observed that the non-dimensional frequencies are close to the value of St = 0.2 within a 10% error shown by dotted lines. Therefore, it can be seen that using the jerk spectrum, as the Von-Karman vortex street Strouhal frequency identifier, holds across the tested range of Reynolds number.

### Size discrimination from the jerk signals

Figure 6h represents the autocorrelation data of the jerk signals from the long whiskers for the 35 mm diameter cylinder for all tested velocities. It can be seen that the first zero crossing occurs approximately at t/T ≈ 0.2, where the delay time (t) is normalized with the time scale of the vortex shedding (T), which is calculated from the Strouhal frequency (Fig. 6d–f). Therefore, the dimensional time scale at the first zero crossing is t ≈ 0.2 T, which is also 0.2 (1/*f*). From Eqn. 1 (1/*f*) is equivalent to ($D/(St\cdot {U}_{\infty })$). By Taylor’s hypothesis, the time scale from the autocorrelation function (t) could be multiplied by the convective flow velocity (*U*_∞_) to obtain the length scale of the vortices^34^, (i.e., $0.2\cdot {U}_{\infty }\cdot D/(St\cdot {U}_{\infty })$) which reveals the upstream model diameter (D), given that the Strouhal number is a constant ($St=0.2$). This result is also consistent for the other tested cylinders and is shown in Fig. 6j,k. Consequently, it can be stated that the whisker’s jerk signals provide a length scale estimate of vortex generating body.

### Characteristic time scales of a whisker motion

**Figure 7:**
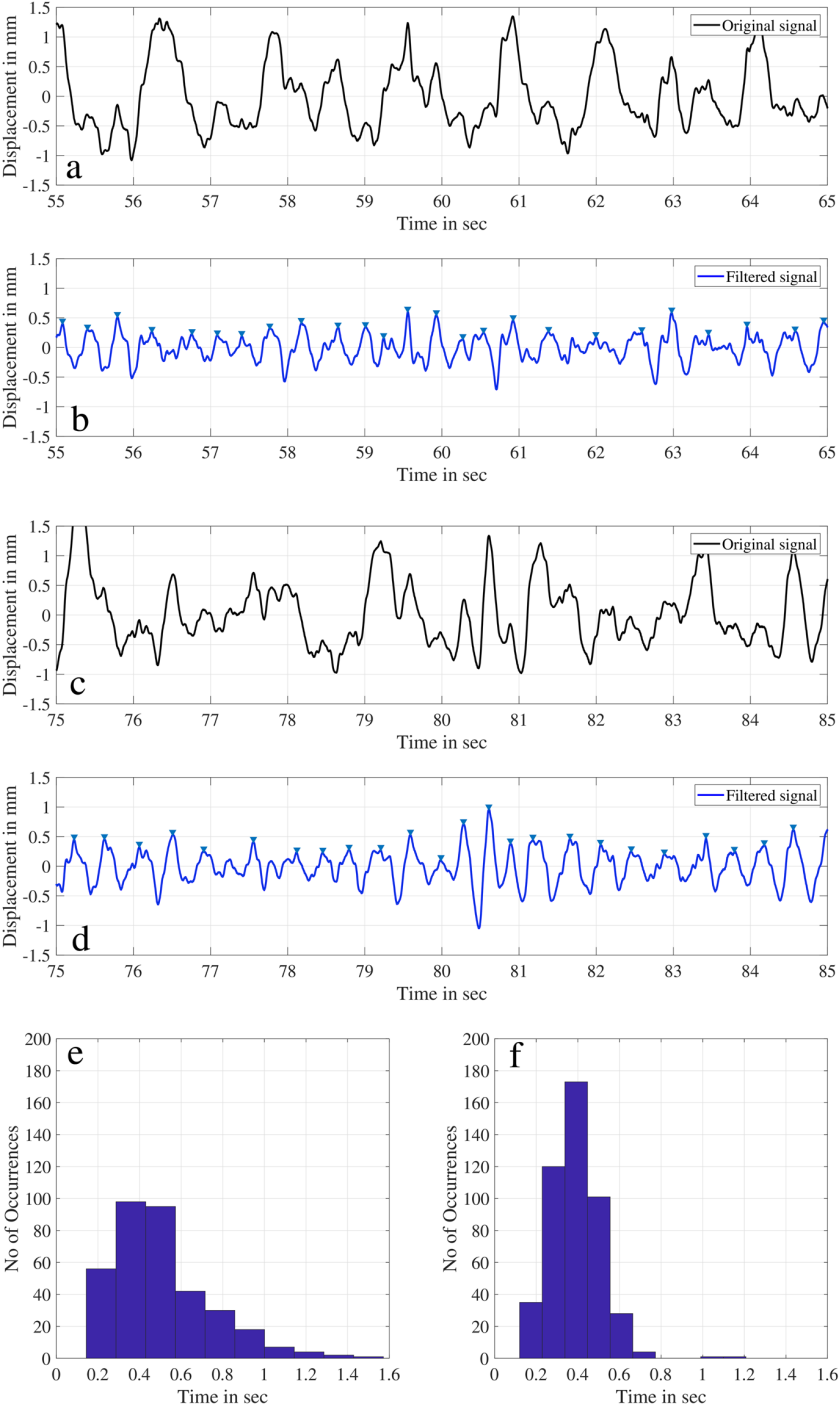
(**a**,**c**) Original signal of whisker-3 (w3) for the time period of 10 seconds. (**b**,**d**) Filtered signal for the same time period. (**e**,**f**) Histogram of peak-to-peak time for original and filtered data.

The displacement time history of whisker (w3) at a velocity of *U*_∞_ = 30 cm/s and in the wake of an upstream cylinder of D = 25 mm is shown in Fig. 7a,c for two 10-second recording intervals. The Strouhal frequency from the Von-Karman vortex street is 2.4 Hz for this condition. The jerk spectrum of this same signal is shown in Fig. 5c and shows a distinct peak at this frequency. Therefore, the time scale of impact between two consecutive vortices of the same sense of rotation is about 0.42 seconds. At the time scale mentioned, we could not recognize the variation in the values from the original signal, but only at a lower frequency corresponding to the first wake sub-harmonic time scale of about 0.8 to 1 second. Because of this, the peak in the bending spectrum plot corresponding to the subharmonic frequency is larger as shown in Fig. 4c. This larger modulation obscures the whisker’s jerky motion in the bending displacement domain. The physical reason for this sub-harmonic will be discussed later. However, when we filter the 1.2 Hz sub-harmonic frequency from the original signal, we can visualize the short jerky kinematic events shown in Fig. 7b,d. This is very evident in the filtered signal where the whisker reverses it’s motion from forward to backward with a time scale equivalent to the Strouhal time scale of 0.42 seconds. This time series is periodic and exhibits about 24 to 25 short kinematic events in time for the two distinct recording intervals shown here. Furthermore, the occurrence of peaks corresponding to the original signal and filtered signal is given as a histogram in Fig. 7e,f for the total recording period of about 180 seconds. The time scale spread of kinematic events for the original signal (Fig. 7e) conveys the information that the jerky short kinematic events cannot be separated from the other flow events. However, the dominant time scale is the one corresponding to the Strouhal time scale from the filtered data (Fig. 7f). These periodic events of jerky movement make the Strouhal frequency the most dominant peak in the jerk spectrum.

### Vortex motion over the sea lion whisker

The passage of successive vortex cores in the Von-Karman vortex street over the whisker defines the vortex shedding frequency from the upstream cylinder. The experiments were repeated with concurrent PIV flow field measurements and whisker tip recordings to better understand the interaction between vortex core dynamics and the induced whisker motion. In these experiments, the 25 mm cylinder was placed at a lateral offset from the body axis (100 mm offset in the ‘z’ direction) and positioned upstream of the sea lion model such that the vortex street faces the whiskers only on one side of the model; the same side that the LED light-sheet illuminated the flow. The vortex rollers shedding from the upstream cylinder are identified as regions of concentrated Q-values^35^ in the measured region. These are sometimes difficult to observe due to the presence of the short whiskers and reflected light from the model which affects the image quality. The ‘Q’ criterion is defined as the second invariant of velocity gradient tensor defined by Eqn. 3, which visualizes the vorticty field (Ω) by subtracting the mean shear (S) from the flow field.

3 $$Q={\Omega }^{2}-{S}^{2}$$

**Figure 8:**
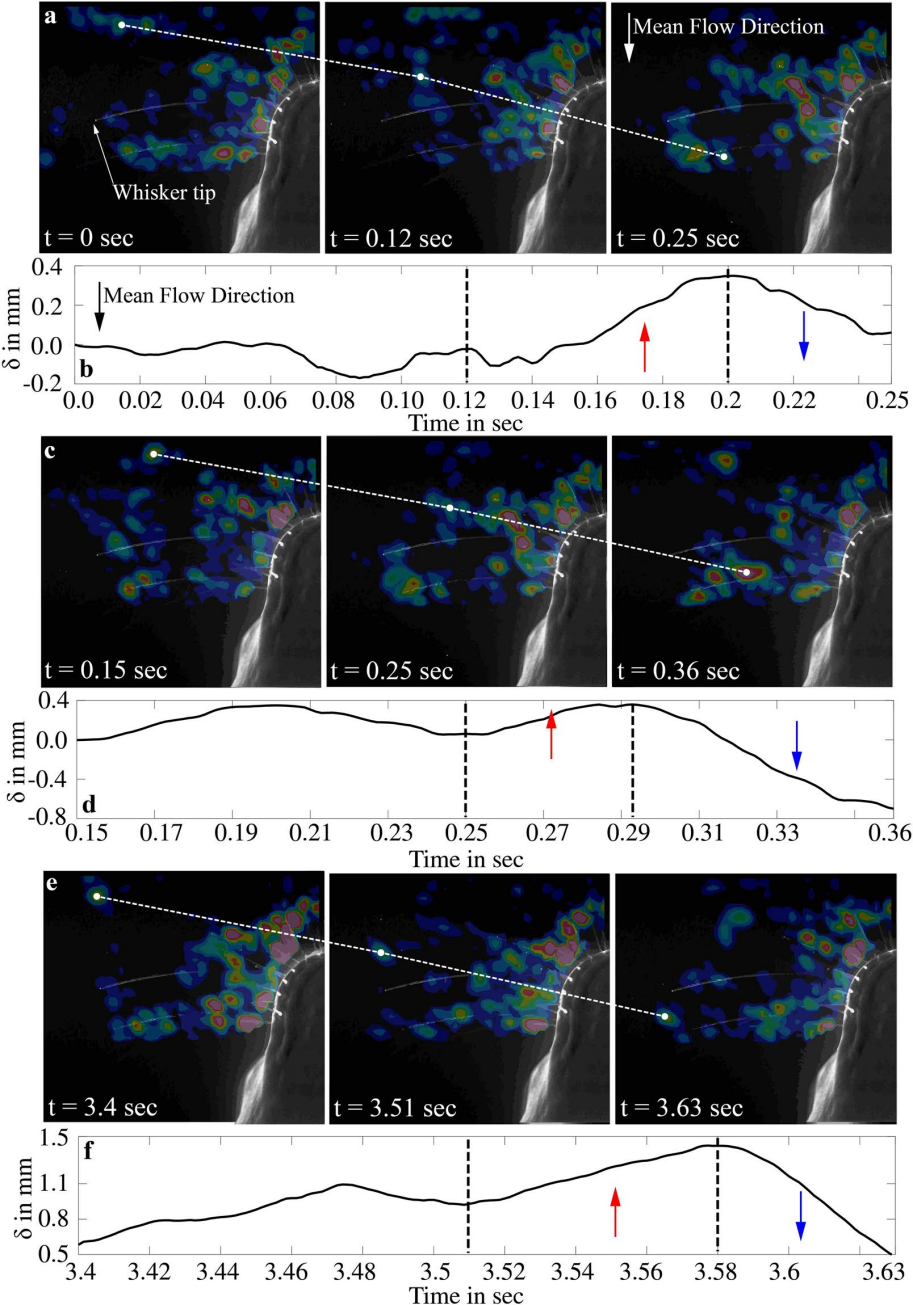
(**a**,**c**,**e**) Represents the Vortex core motion on three time instants. Mean flow direction is indicated in the (t = 0.25 sec of a) Vortex cores are marked with filled white circles. (**b**,**d**,**f**) Represents the displacement (*δ*) of whisker w3 on time scales corresponding to the vortex core motion in (**a**,**c**,**e**). Note: The blue and red arrows in (**b**,**d**,**f**) indicates the motion of the whisker in flow direction and against flow direction respectively.

In the following figures, a series of images were selected where the location of the shed vortices could be tracked over time while crossing whisker w3. In Fig. 8a, a shed vortex from the upstream cylinder approaches the whisker w3, at a whisker length of about 75% from time 0 to 0.12 sec. Initially, there is no significant whisker motion except for minor scale oscillations (see Fig. 8b). However, when the vortex approaches the whisker, the whisker begins to move towards the vortex (the anterior direction). It suggests that the whisker bending due to the mean flow drag relaxes slightly due to the presence of the vortex in front of the whisker. As the vortex crosses over the whisker (as shown in Fig. 8a at time t = 0.25 sec), the whisker reverses its motion (the posterior direction) and eventually follows the vortex downstream until it approaches its initial equilibrium position again. A consecutive shed vortex from the upstream cylinder approaches the same whisker in Fig. 8c at time t = 0.25 sec. As the vortex approaches the front of the whisker, the whisker moves against the mean flow (the anterior direction) towards the vortex and then reverses the direction (the posterior direction) when the vortex passes over the whisker. In Fig. 8d at the time t = 3.4 sec another shed vortex directly passes the tip of the whisker. A similar pattern of forward-backwards motion as previously stated is observed. The observed motion pattern can only be described by the pressure gradient in the vortex core, as the sense of rotation of vortex does not produce a noticeable difference in the observed whisker motion (see Appendix-2). A vortex core in water flows is a concentrated low-pressure region compared to the ambient flow due to angular momentum conservation^36^. This pressure difference is proportional to the $\rho {V}_{\theta }^{2}$, where *V*_*θ*_ is the circumferential velocity within the vortex core. When a vortex, shed from the cylinder and embedded in the mean flow approaches the whisker, the low-pressure region in the vortex core reduces the drag force on the whisker. This, in turn, decreases the net bending moment on the whisker, which enables them to move towards the vortex. On the other hand, the whisker bending increases again when the vortex crosses the whisker as the low-pressure core pulls the whisker in addition to the mean flow drag. These small-scale motions associated with time are repeated at every instant when a vortex core moves across the whisker, and these occurrences define the frequency of the vortex shedding. Note that the sense of rotation of the vortices does not influence this jerky motion, therefore it does not make a difference if the vortex street is generated by the wake of a body (Von-Karman vortex street) or by the propulsion of a fish (inverted Von-Karman vortex street).

### Response of an isolated whisker to a passing vortex street

**Figure 9:**
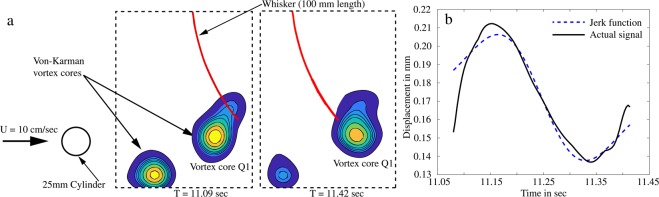
(**a**) Vortex cores from upstream cylinder at two time instants. (**b**) Whisker response corresponding to the vortex passing in the same time frame.

A better understanding of the whisker’s interaction with a vortex core is only possible with an isolated whisker facing the vortex street. Using the Euler-Bernoulli beam dynamics, a theoretical model of the whisker dynamics due to a vortex core motion is derived and provided in Appendix-2. It revealed that the vortex only influences the whisker response, when the distance from the whisker to the vortex core is within one diameter of the vortex upstream and downstream. To verify this, a PIV experiment with a 100 mm long single whisker with an upstream D = 25 mm cylinder at a flow velocity of *U*_∞_ = 10 cm/sec was conducted to generate a Von-Karman vortex street. The turbulence effects are reduced at this low velocity and therefore the vortex cores are better identified with the ‘Q’ criterion mentioned above. Figure 9a shows the illustration of the experiment and the two time frames in which one of the Von-Karman vortex cores was passing over the whisker. Within this time frame, the whisker’s actual response is compared with the calculated theoretical jerk motion equation and plotted in Fig. 9b (see Appendix-2). It is evident from the response that the whisker moves against the mean flow direction when the vortex approaches, and when the vortex passes, the direction of whisker motion reverses. The comparison with the theory is excellent, suggesting that the whisker only reacts when it is in the vicinity range of the vortex core (i.e. one vortex core diameter upstream and downstream).

### Slip stick effect analogy by the whisker response

**Figure 10:**
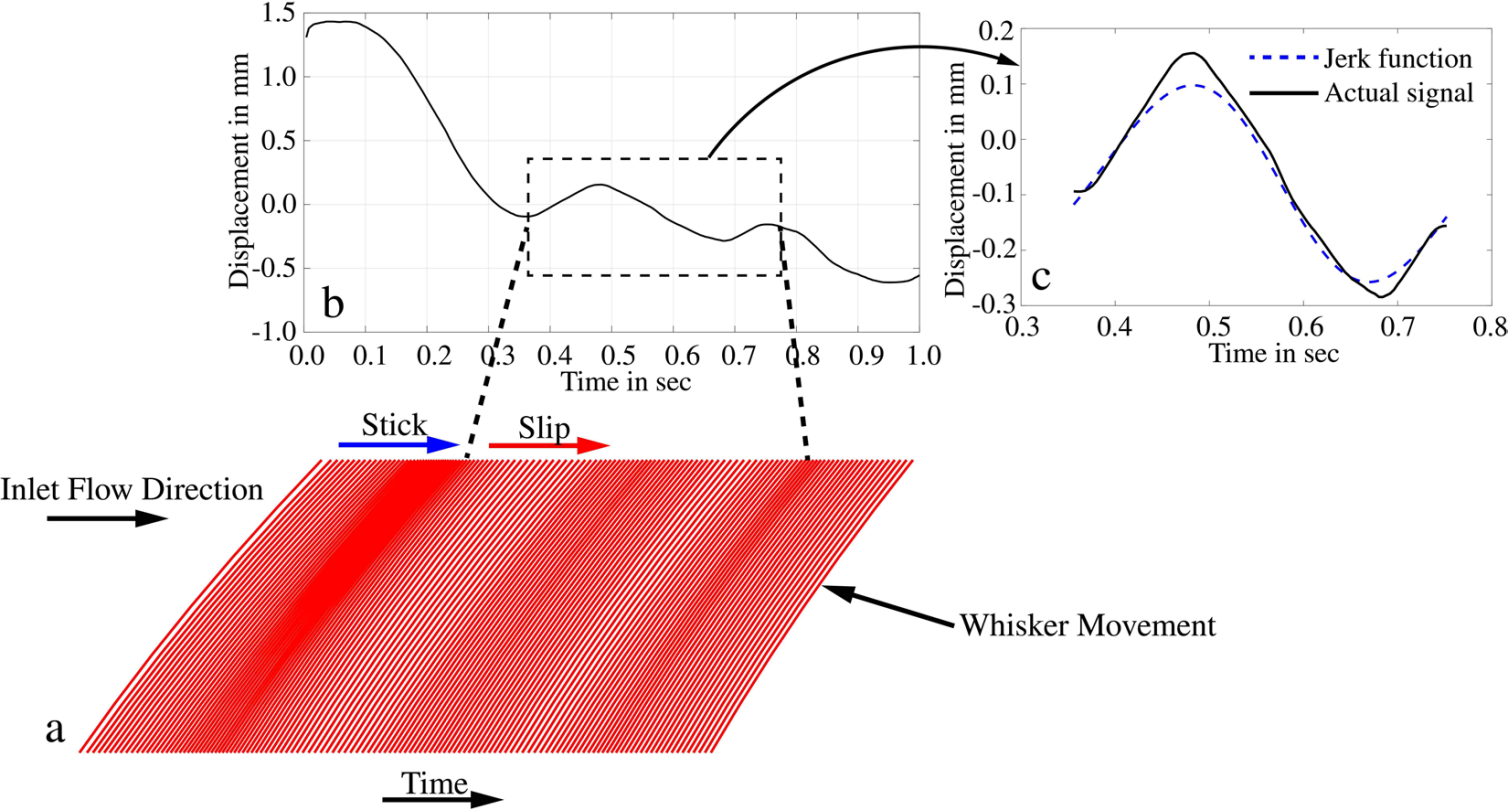
(**a**) Reconstructed whisker deflection at various time instants. (**b**) Displacement signal of whisker w3 at a given time frame with a jerky signal. (**c**) Jerk signal compared with the theoretical jerk function.

The displacement response of whisker w3 for a time frame of 1 second is shown in Fig. 10b. This motion is reconstructed by up-scaling the whisker tip displacement in the time interval and is shown in Fig. 10a. The signal in the time interval from 0.35 to 0.75 seconds represents a jerky motion compared to the theoretical jerk function and shown in Fig. 10c. The whiskers undergo a large motion in the direction of the mean flow from the time interval of 0 to 0.35 seconds. This causes the whisker to bend with the flow and hence is defined as the stick phase. However, when the whisker bending deflection relaxed, defined as a slip phase, the whisker undergoes a mild forward motion between the time interval of 0.35 to 0.5 seconds. Again, the whisker moves in the direction of the mean flow comparable to the stick phase from a time interval of about 0.5 to 0.7 seconds. These effects could be observed by the fringe intensity of red colour lines in Fig. 10a (stick-phase fringe-spacing is denser and slip-phase fringe-spacing is less dense). This illustration is inspired by Fig. 4C of Oladazimi *et al*.^37^ showing a videographic trace of whisker movement. Our reconstruction is analogous to this in a fixed coordinate system when the sea lion passes a vortex street. The stick-slip effect is clearly visible from this reconstruction of the whisker motion.

## Discussion

Our experimental study revealed many interesting outcomes on the pinniped whisker response to Von-Karman vortex streets. The animal’s head alters the flow structure around it considerably, resulting in the amplification of the subharmonic mode in the Von-Karman vortex street. It is strongly supported by the fact that even a small rod in a circular cylinder’s wake will enhance its subharmonic mode^38^. However, all whiskers are effective at identifying the Strouhal frequency with variations only in the peak elevation by using the jerk signal. Secondly, whiskers in front of the snout always display a more defined peak in frequency plots compared to whiskers in the aft portion of the head (see the frequency response obtained from w1, w2 and w3 compared to w4,w5 and w6). It is therefore thought that this could be the reason why seals and sea lion to protract their whiskers in the most forward position to minimize body interference. The time series of whisker motion in Fig. 8 shows that the whisker’s jerky response occurs when a vortex core passes over it. In a vortex core, the pressure variation closely resembles a Gaussian distribution. Therefore, based on the pressure distribution from the vortex core, a theoretical model for the whisker response was developed. It was identified that the whisker response *J*(*t*) is a time derivative of the Gaussian function given by Eqn. 4 from Appendix-2, where *A* is the maximum amplitude based on several fluid and structural parameters, ‘*R*’ is the radius of the vortex core, ‘*C*’ is a constant depending on the normalised position of the vortex impact on the whisker *y*_*c*_/*L* and (−${x}_{o}+{U}_{\infty }\cdot t$) is the convective location of the vortex core. This theoretical function was validated in Figs 9b and 10c with the actual response of the whiskers. While the whisker’s large-scale movements result from the velocity-induced drag forces, the additional small jerky type motion is the response to a single vortex core passing the whisker. The peaks in the jerk spectrum suggested that the whiskers are highly susceptible to the local pressure gradients in the flow. Also, the size of the body in front of the sea lion could be predicted by the autocorrelation information from the jerk signals. This helps the sea lion identify the fish’s frequency and size when following its hydrodynamic trail.

4 $$J(t)=A\cdot \frac{(\,-\,{x}_{o}+{U}_{\infty }\cdot t)}{R}\cdot {\exp }(\frac{-2{(-{x}_{o}+{U}_{\infty }\cdot t)}^{2}}{{R}^{2}})\cdot C(\frac{{y}_{c}}{L})$$

Now let us discuss the similarities between the sensing mechanism between rodents and pinnipeds. Recent research on rat whiskers revealed that the best way for texture coding is through stick-slip motions that are discrete short-lasting kinematic events^37^. These events are noticeable when the motion of the whisker is differentiated in time. Similarly, for sea lions, the surrounding information includes signatures of vortices left behind in the wake of the fish (inverted Von-Karman vortex street^39^). As shown in Fig. 9b, these vortices produce a forward-backwards type of jerky motion. When this jerky signal is reconstructed in the time domain, we could observe the stick-slip response as displayed in Fig. 10a. From these observations, we will redefine the interpretation of the influence of vortices on whiskers in aquatic environments. First, when the seal or sea lion swims, the whisker bends with the flow because of the fluid-induced forces. This is similar to the rat whiskers stick phase, where the friction between the whisker and the surface creates the sticking effect. Second, in rats, the slip-like events are when the whisker moves faster in the direction of whisking than the reference acceleration when whisking in air. Accordingly, when a sea lion swims through a vortex street and interrogates them with their whisker, each vortex core causes the adjacent whisker to move forward against the direction of swimming or ‘to slip’ from the mean direction of bending. The additional force effect causes the whisker to respond quickly in the stick phase when the vortex core has passed the whisker. It is essential to add the fact that whiskers are tactile sensors, which rats and pinnipeds use to identify their surroundings. For a rat, the key information is about the surface texture that provides them with detailed representation and nearby topology. For pinnipeds such as seals or sea lions, the primary source of their prey’s hydrodynamic signatures is the shed vortices, similar to the textures. Therefore, short kinematic events such as stick-slip mechanisms are common to both species, although the behavioural and evolutionary processes are different.

To summarise, this is the first study to explore the interaction of whiskers installed on a model of sea lion head to vortex streets as they occur in nature. Multiple fibre optic cables with comparable mechanical properties to whiskers were used to monitor the whisker tip motion for various Von-Karman vortex streets generated from upstream objects. The tests were performed without interfering with the flow or by using force transducers which increase the complexity of the experiment. This is because the tip deflection is an immediate indicator of the bending moment at the whisker’s root. Furthermore, the collective response of all whiskers in the field of view can be successfully tracked with the use of the ‘Eye motion’ camera. We have identified that the WIV response in the lower frequency range is at least one order more intense than the whisker’s VIV response. It is claimed here that the use of elliptical cross-section whiskers reduce the VIV response and amplifies the SNR. From the PIV results and the derived theory, it is shown that whiskers slip from the bending direction when they encounter a vortex and stick with a higher rate than the slip phase when the vortex passes over the whiskers. This small jerky movement, which occurs in a shorter time scale, enlarges the time derivative domain (acceleration signal), which amplifies the Strouhal frequency peak elevation and increases the SNR by a factor of three.

## Methods

### Model and whisker setup

Scanned data of sea lion body was acquired from Museo delle Scienze, Italy. The digitized data was cleaned and converted into a 3D model using CATIA. On the head of the sea lion, 38 holes of 0.8 mm diameter were modelled on either side of the head on six rows as mentioned in Sawyer *et al*.^28^. The model was 3D printed at City, University of London. Fibre optic cables of 0.75 mm diameter (based on Summarell *et al*.)^40^ were inserted through the holes from the back end and were cut into the required length of the whiskers which ranges from 5 mm to 115 mm as mentioned by Sawyer *et al*.^28^. All the fibres were glued in position at the model surface to provide a fixed cantilever condition. The open ends of the fibres were bunched together and cut to form a flat surface such that they can all be illuminated with a light source outside the water tunnel. Hence, the fibre optic cable acts as a whisker and the movement of the whisker can be tracked with its light at the tip of the fibre. The optical fibres are made of polymethylmethacrylate (PMMA) plastic material which has an elastic modulus value of around 3.5 GPa (from Leal-Junior *et al*.^41^), and the value of the seal whisker is in the order of 4 GPa as mentioned by Subramaniam *et al*.^20^. Since both the geometrical and the mechanical properties of the whiskers are comparable, we expect this model to act as a passive whisker successfully. However, there are some limitations in this study, the fibre is circular in shape when compared to an elliptical section of the sea lion whisker. The diameter is constant for all fibres, and it is constant along the length, however for sea lions the whisker diameter changes throughout the length and the diameter changes for different whiskers.

### Camera setup and data processing

A high-speed camera (ProcImage 500-Eagle high-speed camera) at a sampling rate of 250 Hz was used to track the motion of the whiskers. It had a pixel size of 1280 × 1024 which relates to a physical dimension of 238 × 190 mm. The built-in function in the camera calculates the centroid of a light spot automatically using the barycentre mode. The camera function is explained in Fig. 2a–d. When the surrounding lights are off, and the fibre optic lights are switched on, the camera records the images and automatically does the image binarization to discriminate between the light tips and the surrounding. It then calculates in real-time the centroid of the light spots in pixels for all the visible fibre tips and saves those as an array of the X-Y coordinate data file. The advantage of this method is the recording can last more than 30 minutes in which we only get the whisker motion data, other methods like standard image capturing would exhaust the memory usage of the computer. The extracted data was smoothed in MATLAB and the power spectrum was calculated with a frequency resolution of 0.1 Hz.

### PIV set-up

A 3 mm thick LED light sheet was focused on the region near the long whisker (Whisker 3) portion of the model. Neutrally buoyant particles of 50 *μ*m were introduced into the water tunnel and were continually added until a suitable seeding density was observed for the PIV measurements. The images were captured with Phantom Miro M310 camera at a frame rate of 700 Hz, for a flow period of 4 seconds. Due to the curvature and orientation of the whiskers, the PIV was arranged in a plane such that two whiskers were within PIV plane. The post-processing of PIV images was carried out using Dantec Dynamic Studio software with adaptive PIV algorithm to calculate the velocity with a pair of two successive images and the velocity was averaged with the 3x3 filter. The Strouhal frequency of the upstream disturbance is calculated from the cross velocity spectrum of the wake, and the vortex core was visualised with an absolute ‘Q’ criterion method.

## Supplementary information

Supplementary Information: Appendix 1 and 2
