# Supplementary material for: Seal and Sea lion Whiskers Detect Slips of Vortices Similar as Rats Sense Textures
Source: Sci Rep. 2019 Sep 5;9:12808. doi: 10.1038/s41598-019-49243-5 (PMC6728330; doi:10.1038/s41598-019-49243-5)
Supplement: Supplementary file 1 — Supplementary Information: Appendix 1 and 2 [file 41598_2019_49243_MOESM1_ESM.pdf]

Supplementary Information

Seal and Sea lion Whiskers Detect Slips of Vortices Similar as Rats Sense Textures

Muthukumar Muthuramalingam, Christoph Bruecker

Appendix 1

Von-Karman Vortex Street

When a bluff body (e.g. cylinder, square or triangle) experiences a fluid flow, unsteady separated regions originate from the aft part of the body in an alternating pattern. These swirling vortices are known as Von-Karman vortices (shown in Fig.1) and they are related with a frequency known as vortex shedding frequency or Strouhal frequency [2]. When this frequency is normalised with the diameter (D) and the incoming flow speed (U_∞)the non dimensional number is known as Strouhal number. It is a constant value of 0.2 over the range of flow speeds [2]. The Strouhal number is defined by the Eqn.1.

$$St = \frac{fD}{U_\infty} \tag{1}$$

f - Frequency of the shedding vortices,
D - Diameter of the cylinder,
U_∞ - Incoming flow velocity.

Hence for a given frequency and oncoming flow speed one can to find the size of the object considering the fact that the Strouhal number is a constant.

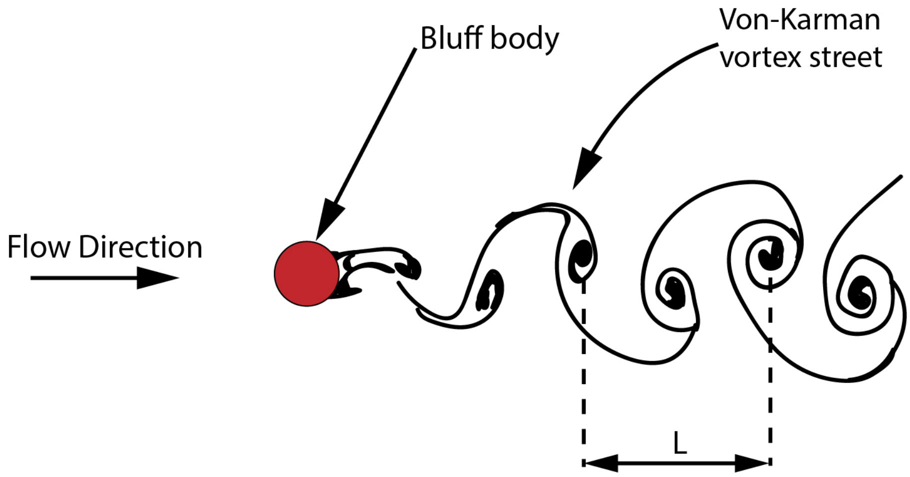

Figure 1: Unsteady flow over cylinder. Adapted from Eloy 2012 [1]

Vortex Induced Vibrations (VIV)

If a body in a fluid flow is allowed to move because of the structural flexibility and the fixation at the support, the body can move in the direction of the force exerted by the fluid flow. If there is no unsteady flow around the body then the displacement of the body will not change with time. However, when a bluff body like a cylinder is placed in a flow, Von-karman vortices shedding from the body will produce periodic time varying forces in the crosswise direction of the flow with a frequency matching the Strouhal frequency explained above. This will lead to periodic oscillations of the body with time, this vibration or oscillation is called the Vortex Induced Vibration (VIV)[2]. The VIV is mostly in direction perpendicular to the mean flow direction as shown in Fig.2.

Wake Induced Vibrations (WIV)

When two bodies are in tandem (one behind another) the wake behind the leading one modifies the dynamics of the fluid flow around the trailing one. Then the displacements of the trailing body will depend on the vortices which are shed from the leading body based on the flow velocity and body

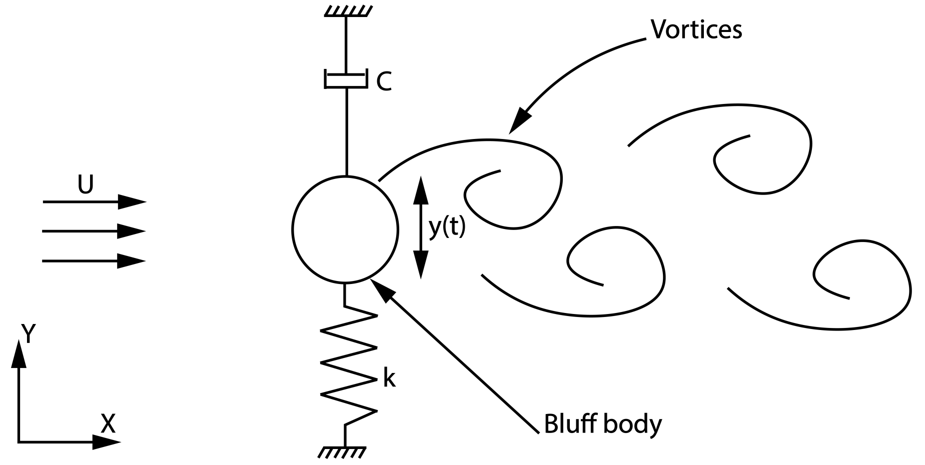

Figure 2: Vortex Induced Vibrations

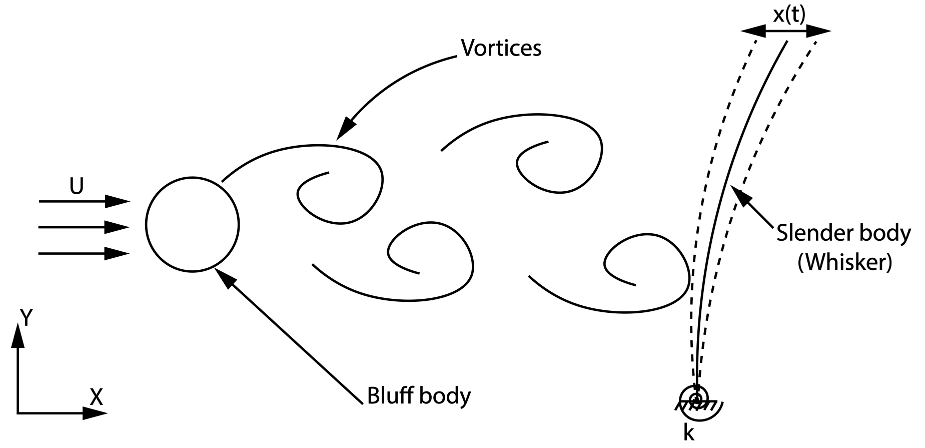

Figure 3: Wake Induced Vibrations

length scale. Hence the influence of the upstream wake dynamics on the displacement response of the downstream body is called as Wake Induced Vibrations (WIV)[2]. WIV depends on the velocity and pressure distribution from the upstream body, material and geometrical properties of the downstream body. For the case of a slender body (whisker) in the wake of a bluff body likea cylinder the WIV are mostly dominant in the direction of the main flow as shown in Fig.3.

Appendix 2

Theoretical model to explain the jerky motion of the whisker as response to a vortex

From the result of VIV in the manuscript (Figure. 3d) it can be observed that the natural frequency of the whisker vibration is about 75Hz. Since it is occuring in the higher frequency range the interaction of the vortex with the whisker can be solved using the quasi-static approximation as the WIV frequencies lie in a low frequency range (less than 3Hz - see Figure. 3c). Consider a fluid flow as shown in Fig.4 which is a combination of an uniform velocity stream (U_∞) in 'x' direction and a vortex with a circulation of (Γ) which creates a rotational velocity V_θ (positive counter-clockwise) from the centre of its core defined by the Eqn.2 [3]. The vortex core is located at $-x_c$ and y_c from the origin which is defined herein at the root (base) of the whisker. The vortex core radius is defined by 'R' and local distance from the centre is given by 'r'. Hence, locally near the vortex, the velocity distribution changes significantly, but with increase in the radial position from the centre the effect of the vortex decreases rapidly because of the exponential decay[3]. Now if we add the fact that the vortex is also moving with the uniform velocity (U_∞) in 'x' direction, then from a coordinate system relative to the vortex core there will be no change in velocity in space. However, for a fixed observer there will be changes in the resulting velocity distribution when the vortex passes through. For example, when the vortex position is exactly at the observer position the resulting streamwise velocity distribution (u(y)) is similar to the plot as shown in Fig.4.

$$V_\theta = \frac{\Gamma r}{\pi R^2} exp(\frac{-r^2}{R^2}) \tag{2}$$

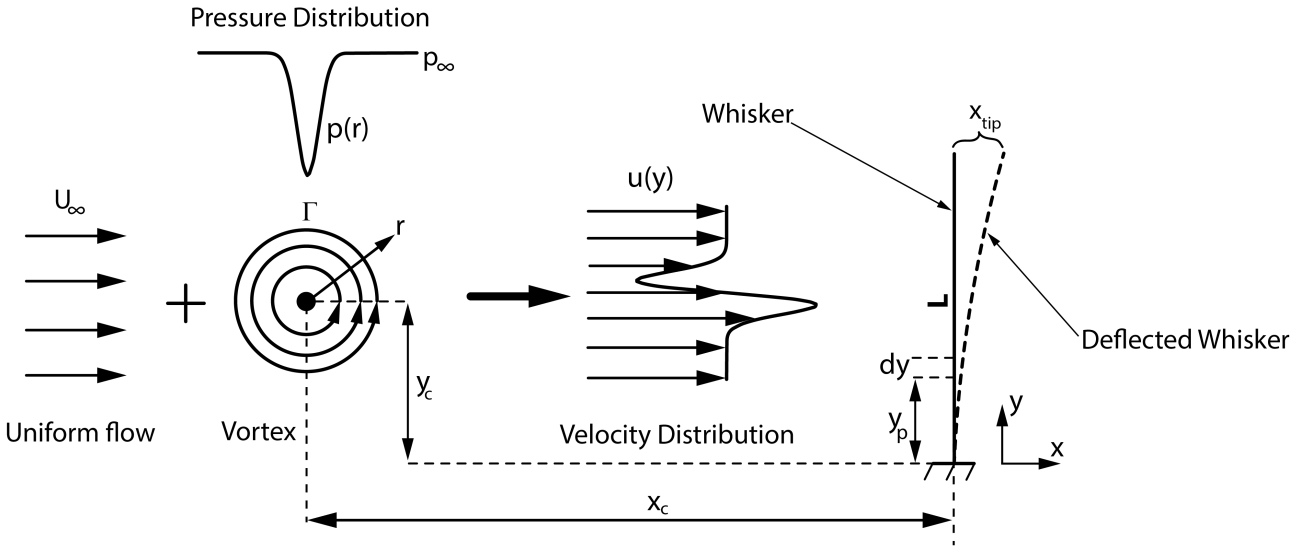

Figure 4: Schematic of the problem

The fluid force acting on the whisker is the summation of pressure forces and drag forces caused by the local velocity distribution. First we will consider the effect of velocity induced drag forces alone. A whisker of length (L) and diameter (d_w) is placed perpendicular to the uniform flow direction. When the vortex is far upstream, the whisker will experience a uniform steady flow, and the load on the whisker remains constant in time. But when the vortex passes over the whisker the load changes with time. The whisker is considered in first approximation as a cantilever beam with its end clamped at the base. We consider a small strip of the whisker 'dy' at a distance 'y_p' from the base along 'y' direction as shown in Fig.4. This small strip will experience a drag force (dD_s) given by the Eqn.3,

where ρ is the fluid density, u(y) is the local velocity and C_d is the drag coefficient of the cylinder[4].

$$dD_s = \frac{1}{2} C_d \cdot \rho \cdot u(y)^2 \cdot d_w \cdot dy \cdot \tag{3}$$

Hence, contribution to the bending moment at the base because of this small strip is given by Eqn.4.

$$dM_D = dD_s \cdot y_p \tag{4}$$

The net bending moment acting on the whisker base is the integral of the moment from root to tip in 'y' direction given by Equation.5.

$$M_D = \int_0^L (dM_D) dy \tag{5}$$

This moment balance equation is solved for all the vortex positions from far upstream to downstream with the use of Finite Element Analysis of a Euler beam theory[5]. The resulting deflection is calculated from the moment distribution on the whisker because of the velocity induced drag forces. For a flow with a circulation value of $\Gamma = 0$ (i.e, uniform flow alone) the deflection at the tip is denoted as $x_{tip@\Gamma=0}$. The 'x' axis is normalised with the vortex core radius 'R' to explain the effect in terms of relative position of the vortex core to the whisker. From Fig.5 it can be observed that the vortex has an effect at 2R distance from the whisker and the tip deflection for both clockwise and counter clockwise direction is not varying more than 1% of the deflection from the uniform flow. It means that the contribution of the vortex induced velocity field on the tip deflection is practically negligible compared to the mean flow velocity.

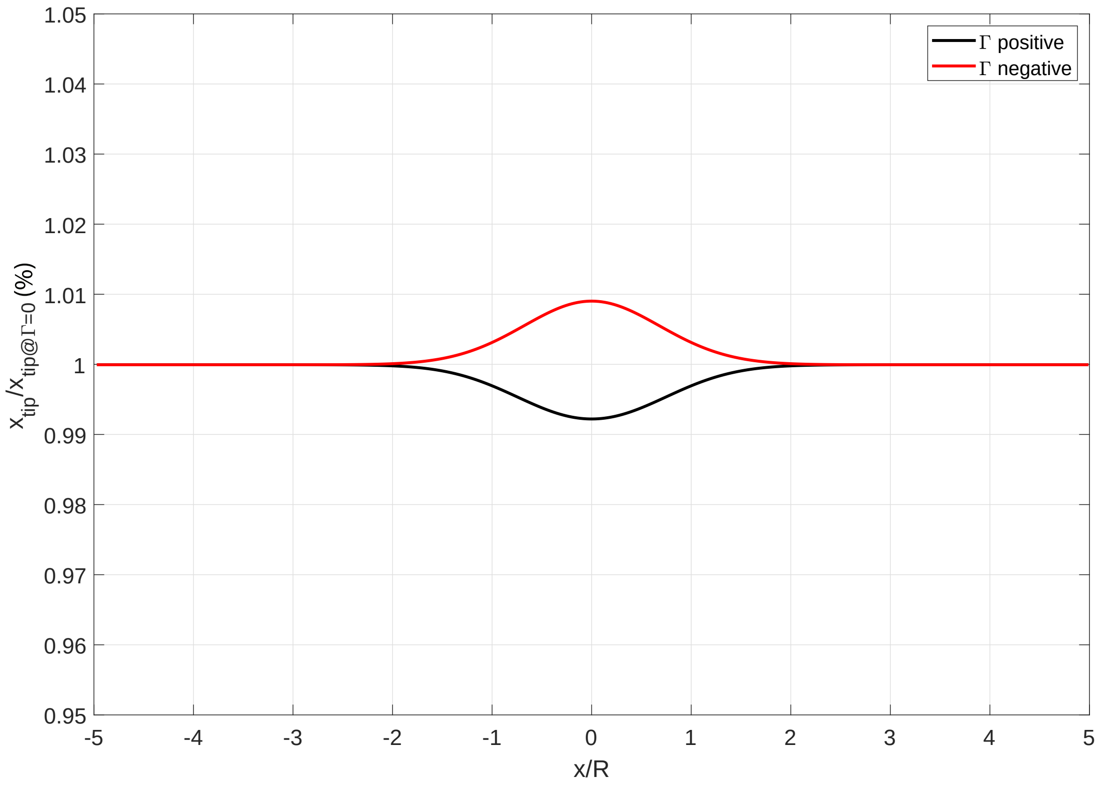

Figure 5: Deflection of whisker with velocity loading

Let us further discuss the moment onto the whisker which is caused by the pressure forces generated by the vortex. The pressure gradient in radial direction is governed by the conservation of angular momentum[4] and given by Eqn.6. Integration of this Eqn.6 will result in the radial pressure

distribution (Eqn.7). It reveals the fact that the local pressure distribution is a function of the circulation Γ with the minimum inside the vortex core and increasing to the free stream pressure at large radius as shown in Fig.4. It is also evident that the pressure is independent of the sign of rotation.

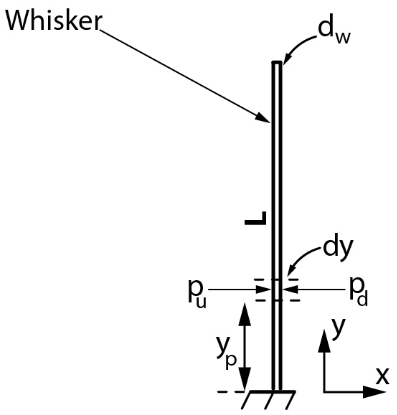

Figure 6: Pressure loading on the whisker

$$\frac{\partial p}{\partial r} = \frac{\rho \cdot V_\theta^2}{r} \tag{6}$$

$$p(r) = \frac{\rho \cdot \Gamma^2 \cdot exp(\frac{-2r^2}{R^2})}{4 \cdot \pi^2 \cdot R^2} \tag{7}$$

$$p(x,y) = \frac{\rho \cdot \Gamma^2 \cdot exp(\frac{-2(x_c^2)}{R^2}) \cdot exp(\frac{-2(y_c-y)^2}{R^2})}{4 \cdot \pi^2 \cdot R^2} \tag{8}$$

The whisker in the flow is now analysed with the pressure loading from the vortex and the pressure distribution is given in Cartesian coordinate system by Eqn.8. A segment of the whisker is considered with a length of dy and at a distance of y_p from the base as shown in Fig.6. The pressure upstream on the segment is denoted as p_u and the pressure downstream is denoted as p_d and they are calculated from Eqn.8. Hence, the pressure force on the small strip is given by Eqn.9 and furthermore the moment from the small strip is calculated as in Eqn.10.

$$dF_p = (p_u - p_d) \cdot d_w \cdot dy \approx \frac{\partial p}{\partial x} dw^2 dy \tag{9}$$

$$dM_p = dF_p \cdot y_p \tag{10}$$

To derive a lumped element force approximation, we consider the pressure induced forces as constant over the core radius in y-direction, which allows to integrate the force distribution dF_p to a point force $\hat{F}_p$ acting at the location y_c. As we know the pressure distribution equation in Cartesian form from Eqn.8, the term $\partial p / \partial x$ could be derived as given in Eqn.11.

$$\frac{\partial p}{\partial x} = -\frac{\rho \cdot x_c \cdot \Gamma^2 \cdot exp(\frac{-2x_c^2}{R^2}) \cdot exp(\frac{-2(yc-y)^2}{R^2})}{\pi^2 \cdot R^4} \tag{11}$$

The integral in the 'y' direction of Eqn.11 is then approximated by a value (2R). This approximation is acceptable since the pressure forces vary only within the radius of the vortex core. The lumped point force is given by the Eqn.12 and the bending moment at the base is given in Eqn.13.

$$\hat{F}_p \approx -\frac{2\rho \cdot x_c \cdot \Gamma^2 \cdot exp(\frac{-2x_c^2}{R^2}) \cdot d_w^2}{\pi^2 \cdot R^3} \tag{12}$$

$$M_p = \int_0^L (dM_p) dy \approx \hat{F}_p \cdot y_c \tag{13}$$

For a cantilever beam, with a point force acting at a distance y_c from the base, the tip deflection (δ) equation is given by Eqn.14, where the EI is the flexural rigidity based on the Modulus of Elasticity (E) and the area moment of inertia (I) of the whisker.

$$\delta = \frac{\hat{F}_p \cdot L^3}{6EI} \cdot (\frac{y_c}{L})^2 \cdot (3 - \frac{y_c}{L}) \tag{14}$$

By substituting the value of force $\hat{F}_p$ from Eqn.12 in Eqn.14 the equation for the tip deflection (δ) can be derived and it is shown in Eqn.15. For this given function, at a constant value of y_c/L, the maximum value occurs at a 'x_c/R' value of 0.5 and the maximum amplitude (A) corresponding to this location is given in Eqn.16.

$$\delta = -\frac{2\rho \cdot \Gamma^2 \cdot d_w^2 \cdot x_c}{\pi^2 \cdot R^3} \cdot exp(\frac{-2x_c^2}{R^2}) \cdot \frac{L^3}{6EI} \cdot (\frac{y_c}{L})^2 \cdot (3 - \frac{y_c}{L}) \tag{15}$$

$$A = \frac{0.0615\rho \cdot \Gamma^2 \cdot d_w^2}{R^2} \cdot \frac{L^3}{6EI} \tag{16}$$

With this maximum amplitude the final solution for the tip deflection is given by Eqn.17.

$$\delta = A \cdot \frac{x_c}{R} \cdot exp(\frac{-2x_c^2}{R^2}) \cdot (\frac{y_c}{L})^2 \cdot (3 - \frac{y_c}{L}) \tag{17}$$

Since the vortex is moving at constant convection velocity (U_∞) along 'x' axis, the position of the vortex core with respect to time (t) can be defined by Eqn.18, where 'x_o' is the position of the vortex core upstream to the whisker at reference time (t=0). Then the tip deflection function in space can be transformed into the time domain (see Eqn.19).

$$x_c = -x_o + U_\infty \cdot t \tag{18}$$

$$\delta(t) = J(t) = A \cdot \frac{(-x_o + U_\infty \cdot t)}{R} \cdot exp(\frac{-2(-x_o + U_\infty \cdot t)^2}{R^2}) \cdot (\frac{y_c}{L})^2 \cdot (3 - \frac{y_c}{L}) \tag{19}$$

This is what we call the jerk motion, J(t) which is induced when a vortex is passing the whisker. This function has the characteristics of the time derivative of a Gaussian pulse.

Figure.7 shows the FEM result for the beam deflection for pressure forces on the whisker compared with the theoretical tip deflection from Eqn.17. It is inferred from the result that the whisker response is similar to the derivative of the Gaussian pressure function. The deflection is about 10% of the whisker deflection at uniform flow which is clearly a larger value when compared with the moment due to drag force alone. Also the whisker response is same for both vortex directions since the pressure is a scalar. when the vortex comes before the whisker because of the low pressure in the front face the whisker loading decreases and goes in the opposite direction to the movement of the vortex. As the vortex passes over it the whisker changes its direction. The displacement effect starts with one vortex core diameter upstream and ends one diameter downstream theoretically.

Relationship between tip deflection and the bending moment at base

From Eqn.13 and Eqn.14 it can be observed that the deflection depends directly on the Force value $\hat{F}_p$ and the location of the impact of the vortex on the whisker by the term y_o/L. Then the deflection at the tip is directly proportional to the moment acting at the base of the whisker by Eqn.20 for a whisker with constant material and geometrical properties.

$$\delta \propto \hat{F}_p \propto M_p \tag{20}$$

From this relation we are arguing that the tip deflection we measure from the experiment is a function of the bending moment at the base.

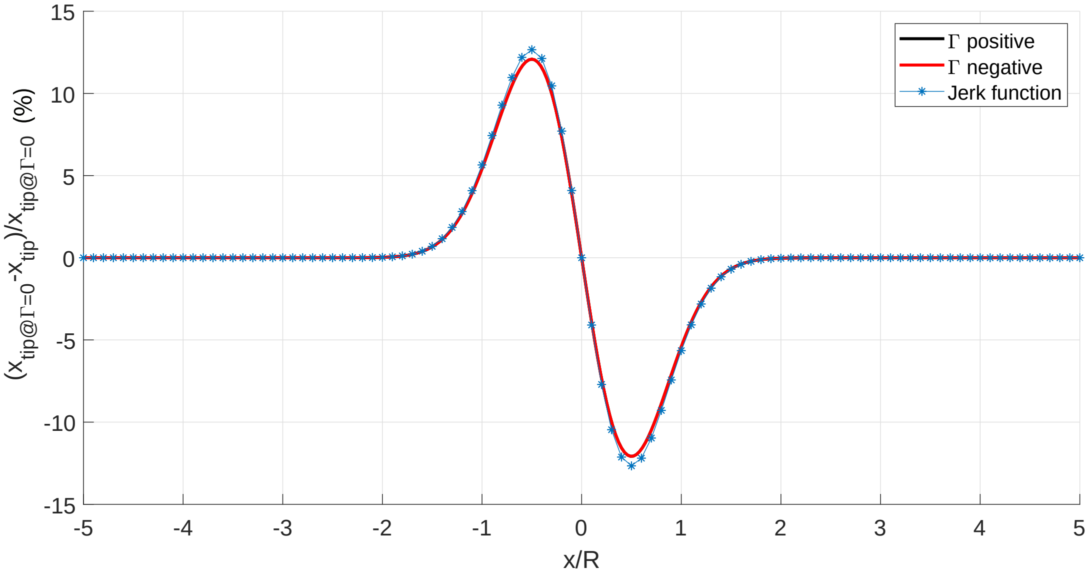

Figure 7: Deflection of whisker with pressure loading

Whisker deflection for various vortex positions and vortex size

Figure.8 shows the whisker deflection for three different cases where the vortex core passes at 30,50 and 70% of the whisker length. It can be observed that as the vortex moves towards the tip the magnitude of the whisker tip deflection increases as the moment arm of the pressure core gets larger from the root and it is immediately observable from Eqn.18 through the term 'y_c/L'. When the vortex diameter is changed relative to the whisker length with constant angular velocity in it, then the magnitude of the tip deflection decreases as seen from Eqn.18 since the radius of the core is in the denominator of the equation. The result is shown in Fig.9. When a whisker is kept very close to the wake generating cylinder then the intense vortices which are generated from the body will produce larger deflection. Similarly, when the whisker is far downstream from the wake generating body, even though the core radius increases because of diffusion the pressure difference is lesser inside the core. This results in smaller deflection.

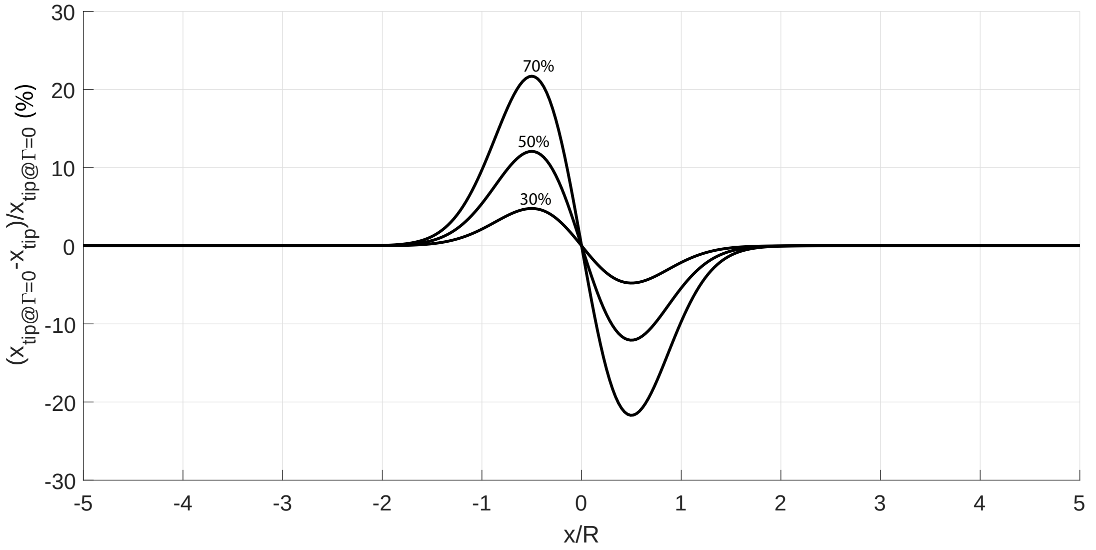

Figure 8: Variation of whisker deflection with position of vortex core on the whisker

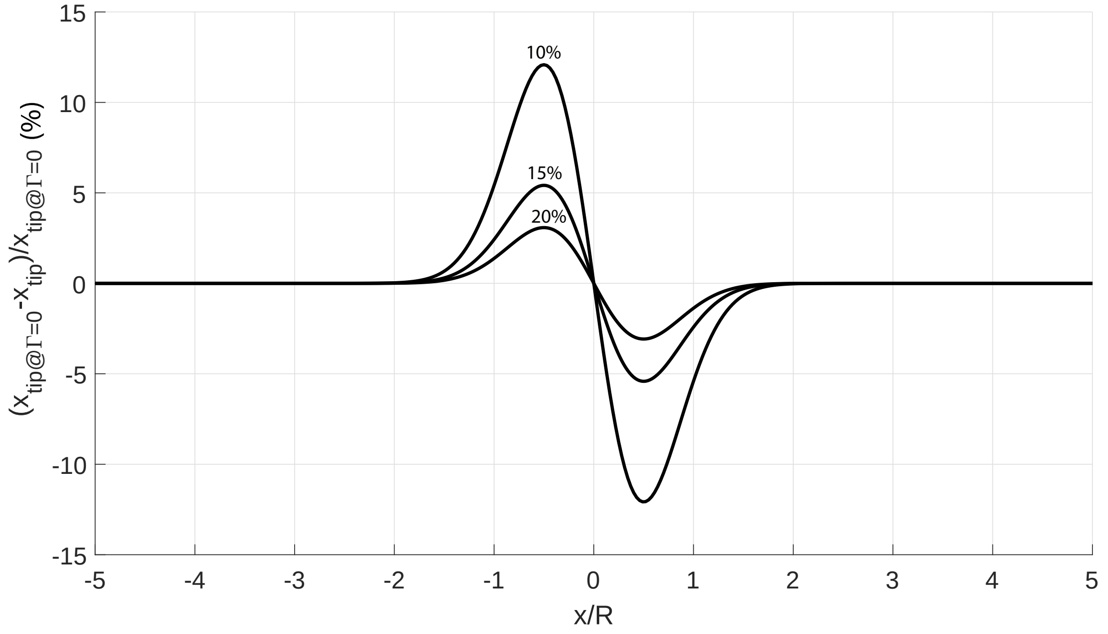

Figure 9: Variation of whisker deflection with vortex core size (constant angular velocity)

References

[1] Christophe Eloy (2012) *Optimal Strouhal number for swimming animals.* Journals of Fluids and Structures **30** pp.205-218.

[2] Heather R Beem and Michael S Triantafyllou (2015) *Wake-induced 'slaloming' response explains exquisite sensitivity of seal whisker-like sensors.* J. Fluid Mech **783** pp.306-322.

[3] Fernando L Ponta (2010) *Vortex decay in the karman eddy street.* Physics of Fluids **22**.

[4] Frank M White(2011) *Fluid Mechanics.* McGraw-Hill **Chapter-3**.

[5] Singiresu S Rao(2017) *The Finite Element Method in Engineering.* Butterworth-Heinemann **Chapter-3**.
